# Prehospital administration of broad‐spectrum antibiotics for sepsis patients: A systematic review and meta‐analysis

**DOI:** 10.1002/hsr2.582

**Published:** 2022-04-01

**Authors:** Joseph Varney, Karam R. Motawea, Omneya A. Kandil, Hashim T. Hashim, Kimberly Murry, Jaffer Shah, Ahmed Shaheen, Joy Akwari, Ahmed K. Awad, Amanda Rivera, Mostafa R. Mostafa, Sarya Swed, Dina M. Awad

**Affiliations:** ^1^ School of Medicine American University of the Caribbean Cupecoy Sint Maarten; ^2^ Faculty of Medicine Alexandria University Alexandria Egypt; ^3^ College of Medicine University of Baghdad Nassiryah Dhi Qar Iraq; ^4^ Barry University Miami Shores Florida USA; ^5^ Medical Research Center Kateb Univeristy Kabul Afghanistan; ^6^ New York State Department of Health New York USA; ^7^ Faculty of Medicine Ain‐Shams University Cairo Egypt; ^8^ Rochester Regional Health/Unity Hospital Rochester New York USA; ^9^ Faculty of Medicine Aleppo university Aleppo Syria

**Keywords:** emergency medicine, prehospital antibiotics, prehospital care, sepsis

## Abstract

**Background and Aims:**

Some studies have suggested that earlier initiation of antibiotics has shown positive outcomes in sepsis patients. We aimed to do a systematic review and meta‐analysis to evaluate the effect of prehospital administration of antibiotics on 28 days mortality and length of stay in hospital and intensive care unit for sepsis patients.

**Methods:**

We formulated a search strategy and used it on search databases PubMed, Scopus, Web of Science, and Embase. We then screened the records for eligibility and included controlled studies, either clinical trials or cohort studies reporting prehospital antibiotic administration for sepsis patients. We excluded duplicates, books, conferences' abstracts, case reports, editorials, letters, author responses, not English studies, and studies with nonavailable full text. Animal and lab studies were also excluded.

**Results:**

The total number of studies identified is 1811, 19 were eligible for systematic review and 4 for meta‐analysis (three cohort and one clinical trial). The total number of sepsis patients in the four included studies in the 28 days mortality outcome was 3523 (1779 took prehospital antibiotics and 1744 did not take prehospital antibiotics). Of 1779 who took the antibiotics, 190 died, and of 1744 who did not take antibiotics, 292 died (95% confidence interval 0.68–0.97, *p* = 0.02).

**Conclusion:**

This meta‐analysis reveals that receiving prehospital antibiotics can significantly lower mortality in sepsis patients compared to patients who do not receive prehospital antibiotics. However, more clinical trials and multicenter prospective studies with high sample sizes are needed to get strong evidence supporting our findings.

## 1 INTRODUCTION

Affecting over 750,000 patients each year in the United States, Sepsis kills more than 200,000 people every year and is further the leading cause of death in critically ill patients. About 10% of hospital admissions are septic shock patients constituting 15% of all patients undergoing sepsis.^1^ Septic shock patients have more than 50% increased risk of death which can be attributed to immunosuppressive drugs, chemotherapy, or the rise of antibiotic resistance. Several antibiotics have been introduced to be used in sepsis and septic shock, such as piperacillin/tazobactam, ceftriaxone, cefepime, meropenem, and imipenem/cilastatin.^2^

Sepsis is an extreme body response to infections, in which an infectious insult prompts a localized inflammatory reaction that then spills over to cause systemic symptoms of fever or hypothermia, tachycardia, tachypnea, and either leukocytosis or leukopenia; furthermore, this severe inflammatory response activates the coagulation pathway producing microvascular thrombi the main cause for sepsis‐associated organ dysfunction,^3^ thus sepsis is a life‐threatening illness and should be well managed. The prehospital care provided to patients by emergency medical services (EMS) personnel has accelerated and improved the quality of care in the emergency department (ED).^3^, ^4^, ^5^ Studies have shown that prehospital sepsis recognition can facilitate treatment and attention in the ED. These patients who get deemed septic alerts will get the required diagnostics and treatment sooner.^4^, ^6^ Retrospective data has suggested that the earlier the initiation of medicine in the ambulance has shown positive outcomes in sepsis patients. The potential exists because at least one‐half of septic patients who arrived at the hospital had favorable early intervention results. Of the patients that seek further medical evaluation and treatment at the ED, approximately 50% of the patients with sepsis arrive by ambulance.^7^, ^8^

Consequently, time to antibiotic therapy (TTA) has become a highly analyzed factor within the quality‐of‐care indicator, with many institutions launching proposals to improve the time to administration numbers.^9^ An important point to consider is that TTA describes administration time from triage time and not from time zero when the infection initially started. Time zero may be biologically more significant but typically is unknown and can vary between patients and infections, ranging from hours to days, depending on the severity of the illness.^10^ In this study, we aim to do a systematic review and meta‐analysis to assess the impact of prehospital administration of antibiotics on 28 days mortality and length of stay in hospital and intensive care unit (ICU) for sepsis patients.

## 2 METHODS

### 2.1 Study eligibility criteria

We included all original studies, cross‐sectional, case–control, and case series that reported prehospital administration of antibiotics for suspected sepsis patients. There were no restrictions on areas where the studies were conducted or the age of patients. We excluded overlapping datasets, books, conferences, case reports, editorials, letters, author responses, not English studies, and studies with non‐available full text. Animal and lab studies were also excluded. The study will be a systematic review and meta‐analysis that will evaluate the effect of prehospital administration of antibiotics on 28 days mortality and length of stay in hospital and ICU for sepsis patients.

PICO criteria for our review will be:

Population: Sepsis patients.

Intervention: prehospital antibiotics.

Comparison: no prehospital antibiotics.

Outcome: 28 days mortality, length of stay in ICU, and length of stay in hospital.

### 2.2 Search strategy

In January 2021, we searched the databases PubMed, Scopus, Web of Science, and Embase as well as references of relevant articles to collect all the relevant studies. Search terms used “Sepsis” [Mesh]) AND “Emergency Medical Services” [Mesh]) AND (“Anti‐Infective Agents”[Mesh]) AND (prehospital antibiotics). All references of included articles for full‐text reading were manually searched for relevant articles. All the search results from the electronic search were collected in a citation file and exported to Endnote X7 (Thompson Reuter) for duplicate deletion. According to the stated criteria, three reviewers independently screened titles, abstracts, and subject headings for eligible publications. In the case of a discrepancy between reviewers, a conversation ensued. If a decision could not be unanimously made, the primary investigator, J. V., decided to include or exclude the study. The risk of bias assessment was done with Cochrane and Newcastle Ottawa scale tools for trials and observational studies, respectively.

Quality assessment: Two authors performed the quality assessment. The randomized studies were assessed by the second version of the Revised Cochrane risk of bias tool for randomized trials (risk of bias 2); evaluation of the risk of bias included seven domains: (1) Randomization process, (2) allocation concealment, (3) blinding of participants, (4) blinding of outcomes, (5) attrition bias, (6) selection of reported results, (7) other bias. We assessed the risk of bias and judged each domain as “low risk,” “high risk,” or “unclear risk.”

Data extraction: All authors extracted the data independently using an online data extraction form. A senior author solved all disagreements. The extracted data included the following domains: (1) summary of the included studies (year, design, country, number of patients), (2) Study outcomes.

### 2.3 Data analysis

We used the RevMan software (5.4) to perform the meta‐analysis; the continuous outcomes were measured as mean difference (MD) and standard deviation (SD), and the dichotomous outcomes as risk ratios (RR) with 95% confidence interval (CI). In case of heterogeneity detected by the *I* ^2^ test over 50%, a random effect model was adopted, otherwise, a fixed‐effect model was used. We used the “leave one out” test to solve the detected heterogeneity. In general; the results were considered significant if the *p* value was less than 0.05.

## 3 RESULTS

**Figure 1:**
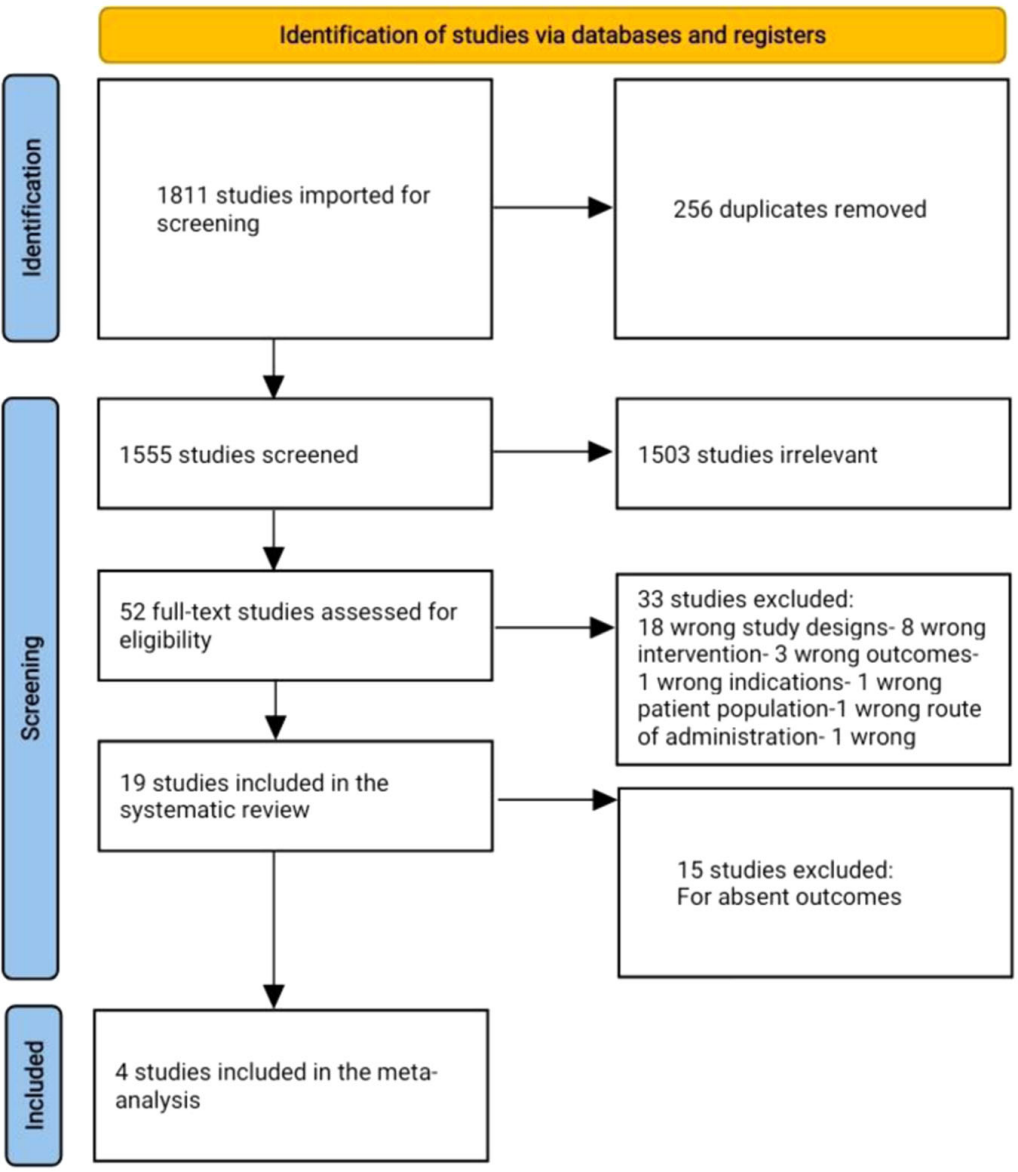
PRISMA flow chart

**Figure 2:**
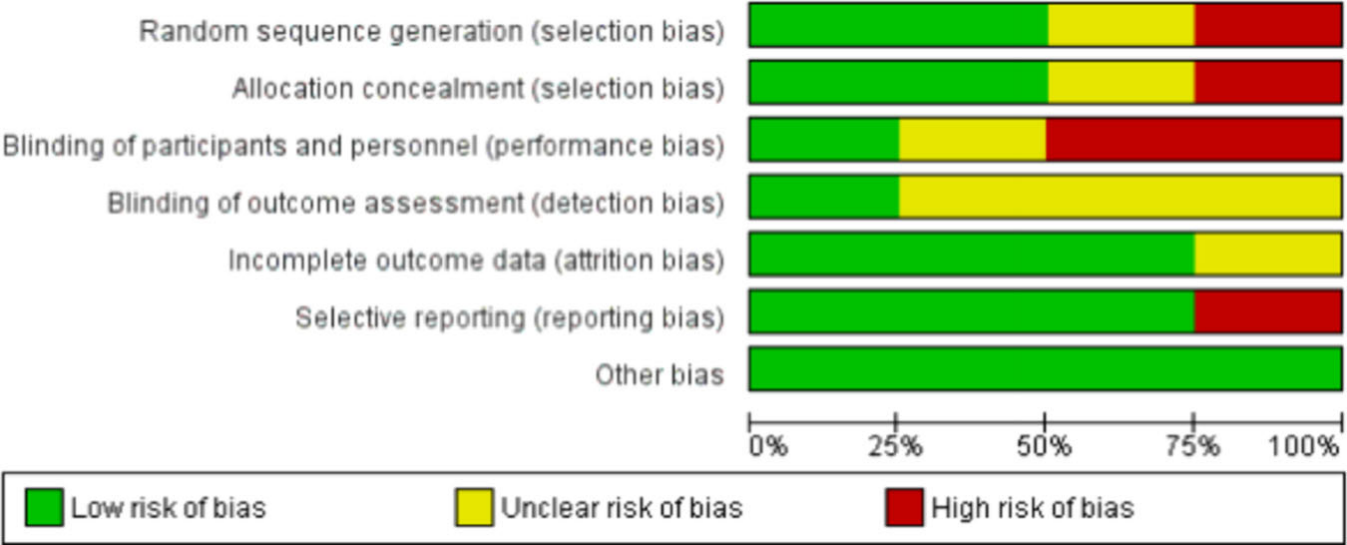
Bias biassessment

**Figure 3:**
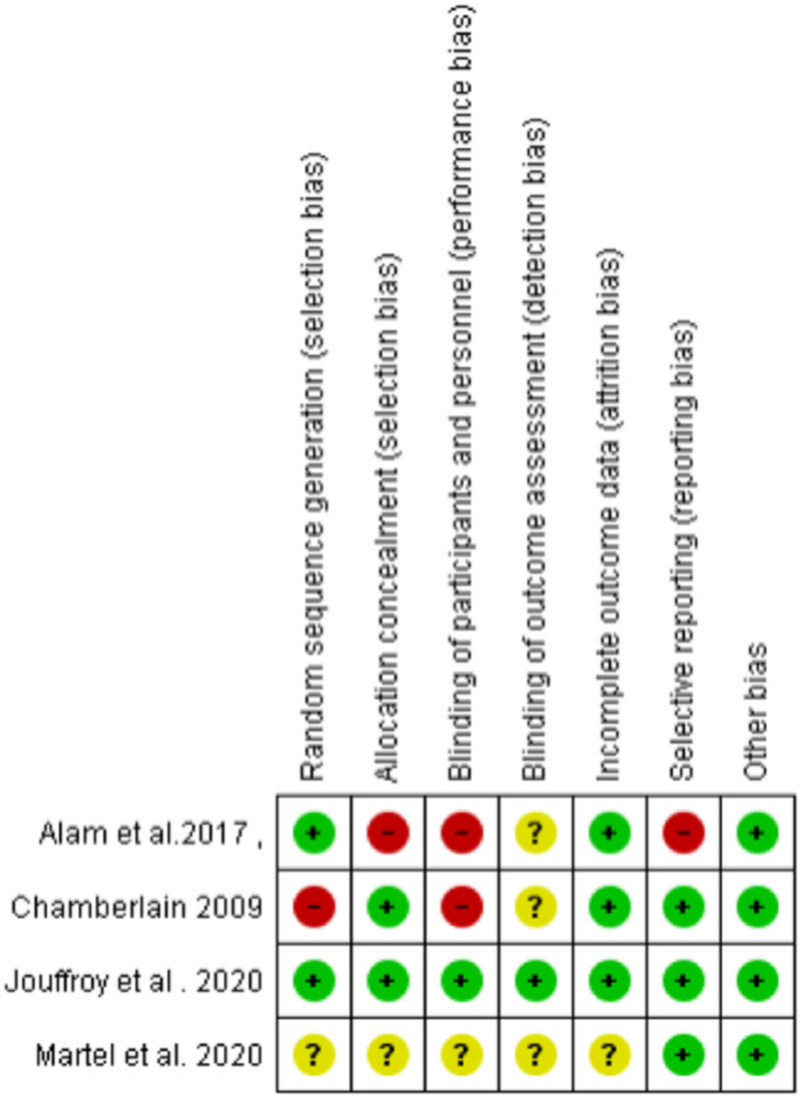
Bias assessment

The complete literature search resulted in 1811 publications for possible inclusion and became 1555 after removing duplications. Of these, 52 were considered appropriate and eligible for a comprehensive review. Nineteen papers were deemed to be eligible for inclusion after complete review and arbitration. Of these, four contained data and were eligible for meta‐analysis (Figure 1). Of the four included studies, four contained data of comparison of 28‐day mortality between patients who took prehospital antibiotics and who did not take prehospital antibiotics, three studies collected data of the length of stay in hospital and ICU admittance in both groups. Table 1 shows the summary of the included studies and Figures 2 and 3 show the two graphs of risk of bias assessment of the included studies in the meta‐analysis. Two studies were of low risk of bias and two were of high risk of bias.

**Table 1:** Characteristics of included studies

The study	Year	Design	Country	No. of patients
Bayer	2013	Retrospective cohort study	Germany	30
Bayer	2021	Retrospective observational study	Germany	56
Chamberlain	2009	Observational and prospective cohort study	Lebanon	127
Chippendale	2018	A prospective study	United Kingdom	113
Cudini	2019	Observational and Descriptive study	Australia and New Zealand	341
Abdallah	2018	A cross‐sectional study	Egypt	100
Jouffroy	2020	Retrospective study	France	308
Khalid	2019	Cohort Study	United States (Pennsylvania)	40,551
Latten	2018	Cross‐sectional study	Netherland	2452
Liu	2017	Retrospective study	United States (California)	51,120
Mikkelsen	2019	Retrospective study	Denmark	117
Nannan Panday	2020	Randomized controlled, open‐labeled trial	Netherland	2658
Rossouw 658	2011	Cross‐sectional study	Netherland	125
Rossouw 659	2011	Retrospective study	South African	605
Sarr	2016	Retrospective study	Gambia	253
Secka	2019	Retrospective study	Gambia	411
Uzodimma	2013	A prospective study	Lagos	100
Martel	2020	A retrospective observational study	United States	347
Joynes	2016	A retrospective observational study	Australia	67

**Figure 4:**
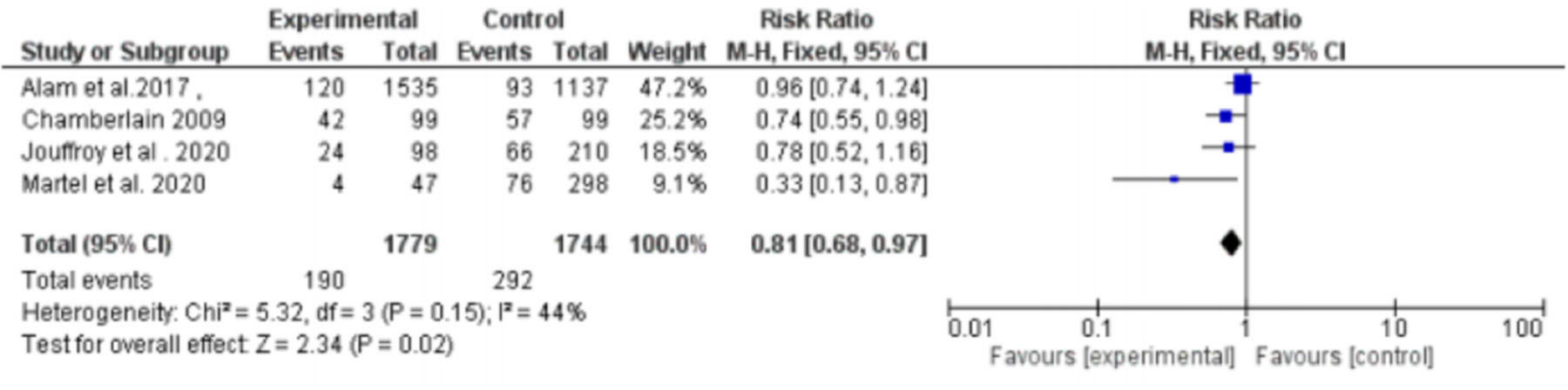
Forest plot of 28 days mortality outcome

The total number of the included patients who received prehospital antibiotics in the meta‐analysis is 1779 (mean age is 72.23 years and 59.19% males). The total number of patients who did not receive antibiotics is 1744 (mean age is 68.45% and 58.66% males). The total number of sepsis patients in the four included studies in the 28 days mortality outcome was 3523 (1779 took per‐hospital antibiotics, and 1744 did not take prehospital antibiotics). Of 1779 who took the antibiotics, 190 died, and of 1744 who did not take antibiotics, 292 died. The pooled RR for patients who took prehospital antibiotics was 0.81 (95% CI: 0.68–0.97, *p* = 0.02) compared to those who did not take prehospital antibiotics. We found no statistically significant heterogeneity (*p* = 0.15), so the fixed effect was used, as shown in (Figure 4).

**Figure 5:**
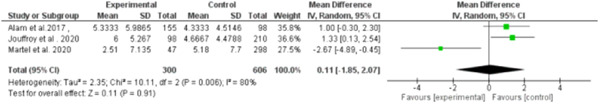
Forest plot of length of stay in intensive care unit after analysis with random effect

The total number of sepsis patients in the three included studies in the length of stay in ICU outcome was 906 (300 took prehospital antibiotics, and 606 did not take prehospital antibiotics). Analysis with random effects was done because we found statistically significant heterogeneity (*p* = 0.006). The pooled MD for patients who took prehospital antibiotics was 0.11 (95% CI: −1.85 to 2.07, *p* = 0.91) compared to those who did not take prehospital antibiotics. We also found statistically significant heterogeneity (*p* = 0.006), which was not solved using random effects, as shown in Figure 5. We did sensitivity analysis with a leave one out test. We removed the (Martel et al.^11^) study, so the heterogeneity was solved (*p* = 0.71). After postsensitivity analysis, the pooled MD for patients who took prehospital antibiotics was 1.18 (95% CI: 0.30–2.06, *p* = 0.91) compared to those who did not take prehospital antibiotics.

**Figure 6:**
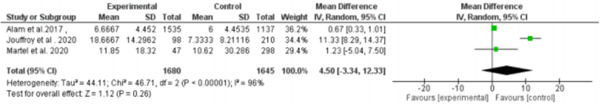
Forest plot in length of stay in hospital outcome after analysis with random effects

The total number of sepsis patients in the three included studies in the length of stay in hospital outcome was 3325 (1680 took per‐hospital antibiotics, and 1645 did not take prehospital antibiotics). Analysis with random effects was done because we found statistically significant heterogeneity (*p* < 0.00001). The pooled MD for patients who took prehospital antibiotics was 4.50 (95% CI: −3.34 to 12.33, *p* = 0.26) than those who did not take prehospital antibiotics. We also found statistically significant heterogeneity (*p* > 0.00001), which was not solved using random effects, as shown in Figure 6. We did sensitivity analysis with a leave one out the test. We removed the (Jouffroy et al.^12^) study, so the heterogeneity was solved (*p* = 0.86). After postsensitivity analysis, the pooled MD for patients who took prehospital antibiotics was 0.67 (95% CI: 0.33–1.01, *p* = 0.0001) compared to those who did not take prehospital antibiotics.

## 4 DISCUSSION

Sepsis has been recognized as one of the most leading causes of death in the past decades. Yet, recently its mortality rates have been significantly decreased as evidence confirms lower mortality rates of PHANTASi by 4% than the previous cohort study by Quinten et al. in the ED.^13^ The sepsis‐associated mortality rates have been linked to the time of receiving antibiotics. Although Seymour et al. reported that the more time septic patients had received broad‐spectrum antibiotics, the more significant risk‐adjusted mortality (odds ratio [OR]: 1.04 per hour delay, 95% CI: 1.03–1.06).^14^ A multicentre retrospective study that included almost 18,000 patients showed that every hour delayed in treatment increased in‐hospital mortality of sepsis patients.^2^ The rapid administration of antibiotics for septic shock has demonstrated improved outcomes, preferably within 1 h after arrival at the ED. A longer duration of treatment has shown worsened outcomes.^15^ The timely administration of antibiotics has been one of the main cornerstones of sepsis treatment.

Burnham et al. showed that even in patients receiving antibiotic therapy rapidly for septic shock, there was still a significant increase in mortality rates.^16^ The author attributed this increase to the main predictor of patient health status, cardiovascular and cellular dysfunction, and not antibiotic therapy timing. A study of over 500 sepsis patients who received antibiotic treatment within 12 h of blood culture showed no difference in mortality rates based on antibiotic administration time. Instead, the only discernible factor for survival was the severity of the sepsis per patient.^16^

The opportunity to identify and deliver the immediate life‐saving antibiotic treatment for septic patients begins at the prehospital emergency level of care; thus, there is an increasing approach to provide the EMS personnel with the needed training to recognize and treat septic patients presented different levels of severity. In addition, Paramedics received additional training to recognize sepsis using screening tools and blood cultures and provide IV antibiotics at the most needed time, as the primary focus of sepsis treatment is to emphasize the immediate delivery of IV antibiotics and oxygen therapy.^15^, ^17^

Administrating antibiotics before patient transport, paramedics giving antibiotic therapy 90 min earlier can potentiate antibiotic's effects more than do current practitioners in metropolitan centers. Therefore, In the Surviving Sepsis Guidelines, it is stated that treatment needs to begin immediately in patients who present with sepsis and meet criteria.^18^ The UK Sepsis Trust has suggested that sepsis treatment starts within 1 h of symptom recognition.^1^ The relationship between each hour delay in treatment and positive patients' outcomes was a nearly linear model.^5^

However, as broad‐spectrum antibiotics such as carbapenems and quinolones seem to be the most common choice, the overuse of antibiotics and antimicrobial resistance should be considered and should be minimized.^19^ Antimicrobial resistance is a global problem with not only clinical but also ethical implications. Clinically antimicrobial resistance leads to lower drug efficacy and higher tolerance. Ethically, as many would argue, whether to prescribe an antibiotic for a patient and face antibiotic resistance yet classify not prescribing an antibiotic as an unethical action.

The timing of antibiotic administration has been unclear. A systematic review and meta‐analysis recently published showed that no significant mortality decrease was seen when antibiotics were administered within 3 h of emergency room sepsis triage or 1 h of recognizing septic shock.^20^ Furthermore, another study reported no association between the administration of antibiotics timing and patient outcomes when suspected sepsis is reported 6 h before presentation at ED.^21^

In our systematic review and meta‐analysis, we reported a 28‐days mortality rate and length of hospital and ICU stay outcomes among sepsis patients who received prehospital antibiotics and did not receive prehospital antibiotics. The pooled data from the included articles showed a statistically significant association between patients receiving prehospital antibiotics and reduced 28‐days mortality rates compared to those who did not take prehospital antibiotics, with no considerable heterogeneity found among the papers. There was no significant effect of prehospital antibiotics on length of stay in hospital or length of stay in ICU unit in both groups.

Early administration of antibiotics has been found favorable for reducing mortality linked in a positive association mostly in patients with a different critical level of illness and a TTA of more than 5–6 h.^14^, ^22^ Studies have also indicated that early antibiotic administration has led to lower patient mortality and a lower prevalence of sepsis progression from severe sepsis to septic shock.^21^, ^22^ Within 28 days, 120 patients had died in the intervention group and 93 in the usual care group. The death numbers increased with increasing sepsis severity in both groups, but no substantial differences were found in the two groups compared to each other. For patients in the usual care group, a longer TTA was not connected with an increase in 28‐day mortality.^10^

In one prospective study, the EMS personnel were trained in sepsis recognition, obtaining blood cultures, and treating the patients with a broad‐spectrum antibiotic, meropenem. The EMS team was directed to administer the antibiotic to “red flag” sepsis patients.^23^ Once trained, EMS was able to identify sepsis patients presenting with “red flag” symptoms at a rate of over 94% accuracy (confirmation was completed by the hospital that received the patient). Blood culture was also found to be adequately done after EMS training, with only 7.1% of the cultures being contaminated, roughly the same as the percentage for hospital‐acquired blood cultures.^22^ Another study found that more gram‐positive bacteria were found within the intervention group, indicating a higher contamination risk of blood culture analysis in the prehospital setting.^10^ Thus, the effectiveness of antibiotic administration and blood culture collection by prehospital providers are still not proven, with further training being warranted.

Patient compliance must also be considered when it comes to prehospital drug administration. Interestingly, 100% of the sepsis alert patients that the EMS attempted to administer the antibiotic to were compliant.^23^ This potentially shows a high trust for the EMS crews, though they do not have the extensive training a physician has for diagnosis and treatment. Infection in a similar study was unable to be confirmed in over 20% of patients.^5^ A more recent study showed that the EMS could diagnose with only 5.3% of diagnosed patients being found to be false positive.^23^ When EMS has quickly and accurately diagnosed sepsis, it has led to faster clinical care, which includes antibiotic treatment.^4^, ^8^, ^24^ This would suggest an overdiagnosis of sepsis by EMS crews. Though not universally accepted yet, there have been attempts to create and implement screening tools that should optimize the diagnosis and treatment of suspected septic patients.^25^ The Sepsis‐3 international task force has proposed a sepsis screening tool, the quick‐sequential organ failure assessment (SOFA) score. Three clinical parameters define the quick‐SOFA score to assess for organ dysfunction associated with infection. These are altered mentation (glascow coma scale < 15), systolic blood pressure ≤ 100 mmHg, and a respiratory rate of ≥22. Two out of three of these criteria are present, and the patient is considered potentially septic if they are SOFA “positive.”^26^

## 5 LIMITATIONS

Our study is limited by the few studies included in the meta‐analysis. Only four studies with 3523 patients in both groups were formed. Some studies did not differentiate between confirmed and suspected sepsis patients in terms of outcomes, so these studies were excluded. Also, one clinical trial is included, and the other included studies are observational. Two studies of the four studies included in the analysis were of high risk of bias. Antibiotics administration may affect the culture results of blood taken upon arrival. Antibiotics given may be inappropriate and increase the mortality risk of sepsis patients. The correct identification of sepsis by the prehospital healthcare team must be taken into consideration. The treatment is only good if the diagnosis is made quickly and accurately.

## 6 CONCLUSION

Our meta‐analysis reveals that receiving prehospital antibiotics can significantly lower mortality in sepsis patients compared to patients who do not receive prehospital antibiotics. However, more clinical trials and multicentre prospective studies with high sample sizes are needed to get strong evidence supporting our findings.

## AUTHOR CONTRIBUTIONS

**Joseph Varney**: conceptualization; data curation; methodology; project administration; supervision; validation; writing—original draft; writing—review and editing. **Karam R. Motawea**: conceptualization; data curation; formal analysis; methodology; project administration; supervision; validation; writing—original draft; writing—review and editing. **Omneya A. Kandil**: data curation; methodology; writing—review and editing. **Hashim T. Hashim**: data curation; methodology; writing—review and editing. **Kimberly Murry**: data curation; methodology; writing—review and editing. **Ahmed Shaheen**: data curation; methodology; writing—review and editing. **Jaffer Shah**: data curation; investigation; methodology; project administration; writing—review and editing. **Joy Akwari**: data curation; methodology; writing—review and editing. **Ahmed K. Awad**: data curation; methodology; writing—review and editing. **Amanda Rivera**: data curation; methodology; writing – review and editing. **Mostafa R. Mostafa**: investigation; writing—original draft; writing—review and editing. **Sarya Swed**: data curation; investigation. **Dina M. Awad**: data curation; methodology; supervision; writing—original draft; writing—review and editing.

 All authors have read and approved the final version of the manuscript. Jaffer Shah had full access to all of the data in this study and takes complete responsibility for the integrity of the data and the accuracy of the data analysis

## CONFLICTS OF INTEREST

The authors declare no conflicts of interest.

## Data Availability

All data generated or analyzed during this study are included in this published article and its supplementary information files.
